# Supplementary figures and images for: Building a mechanistic mathematical model of hepatitis C virus entry
Source: PLoS Comput Biol. 2019 Mar 18;15(3):e1006905. doi: 10.1371/journal.pcbi.1006905 (PMC6445459; doi:10.1371/journal.pcbi.1006905)

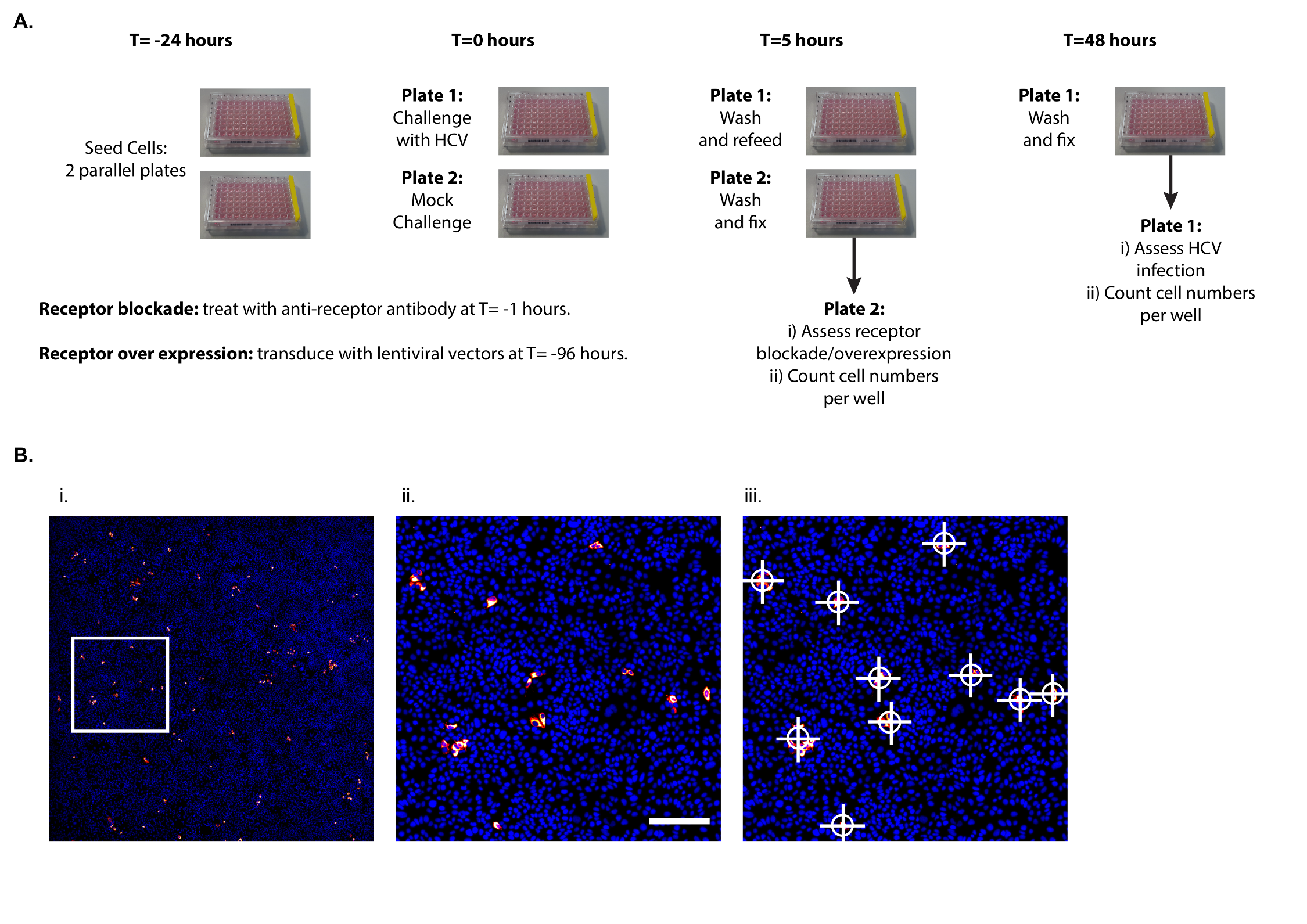

Supplement: S1 Fig — A. The infection assay workflow: cells were manipulated to achieve receptor blockade or over-expression at the indicated time points. Plate 1 was used to determine HCV infectivity, whereas the parallel plate 2 was used to assess receptor blockade/over expression. B. Infection was quantified manually by counting infected foci at 48 hours. At this time point only one round of infection is apparent. i. An example field of HCV infected Huh-7.5 cells stained for viral antigen and cellular nuclei. ii. A large image of the inset from A., multiple distinct foci of infection are apparent. iii. Viral infection was quantified by manual scoring of individual foci, as annotated on to the image. Scale bar 200μm. (TIF) [file pcbi.1006905.s001.tif]

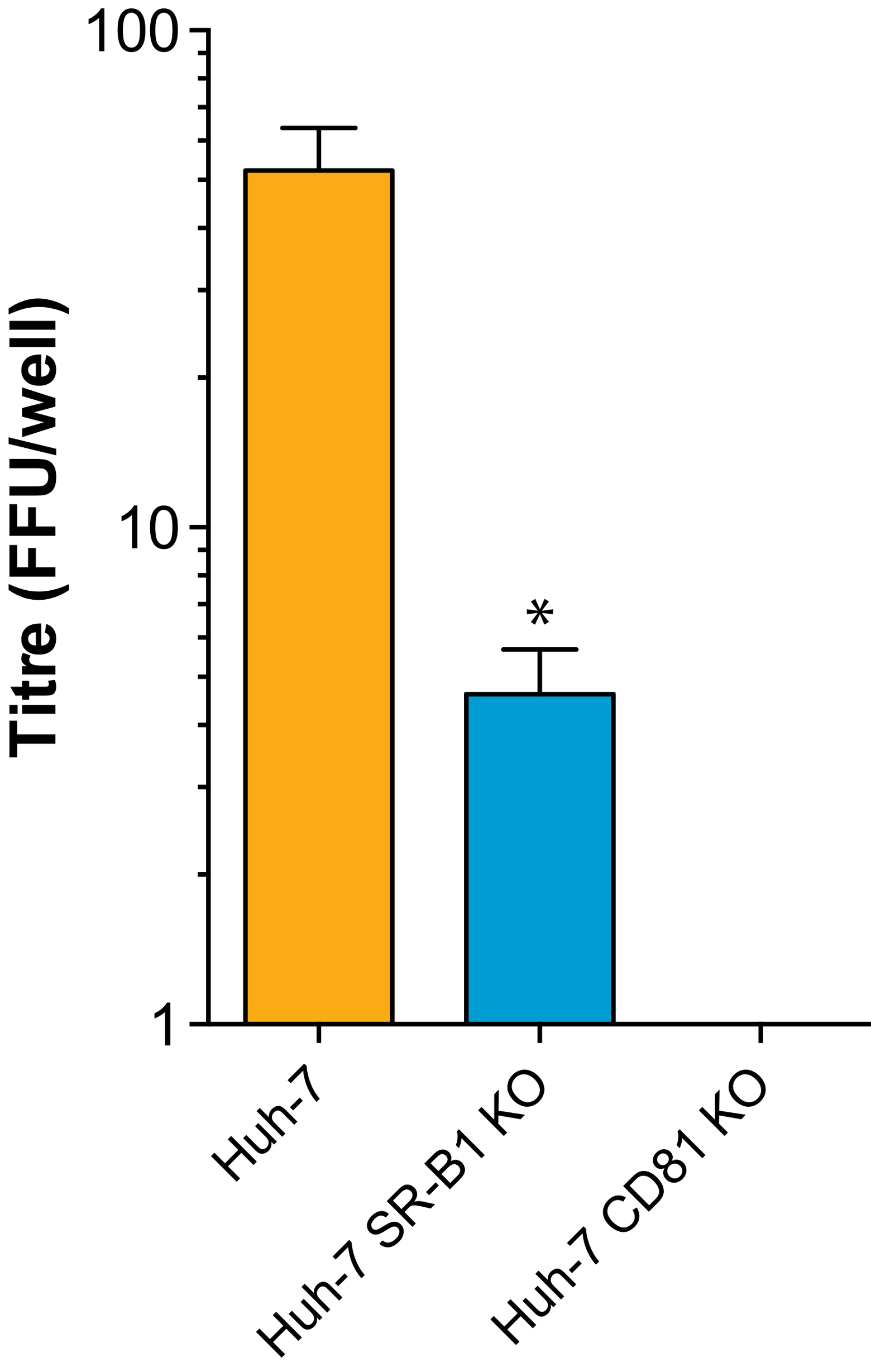

Supplement: S2 Fig — HCV titre in parental Huh-7 human hepatoma cells, or those in which receptor encoding genes have been knocked out by CRISPR Cas9 editing. Mean values of n = 3 independent experiments are shown. Error bars indicate standard error of the mean. Asterisk indicates a significant difference between SR-B1 KO and parental Huh-7 cells (unpaired t-test, GraphPad Prism). (TIF) [file pcbi.1006905.s002.tif]

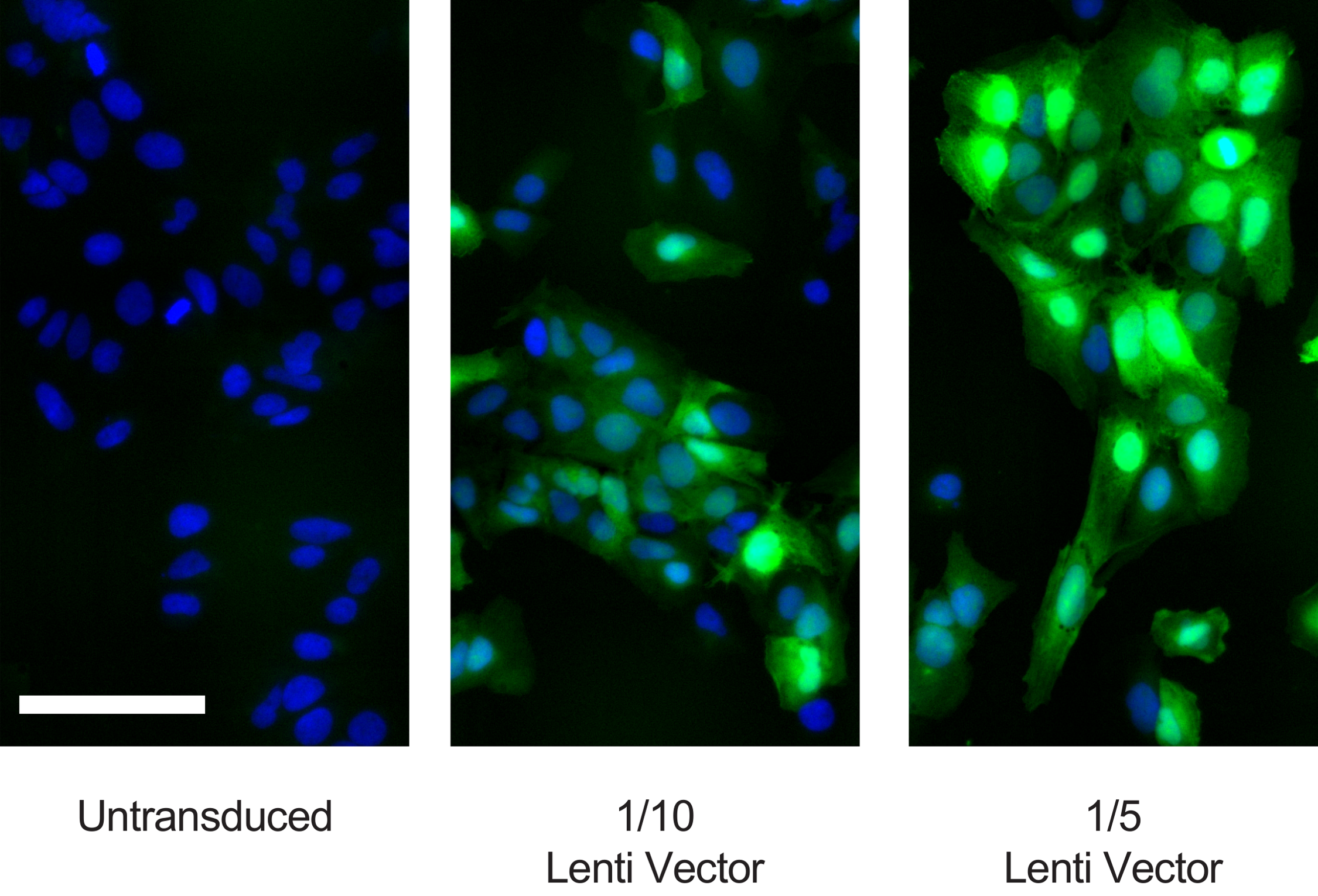

Supplement: S3 Fig — Huh-7.5 cells were transduced with lentiviral vectors that encode both a receptor (either SR-B1 or CD81) and GFP, expressed from separate promoters. Therefore, evaluating GFP expression provides an independent measure of transduction efficiency. The images display representative fluorescent micrographs of parental cells or those transduced with SR-B1 + GFP lentiviral vectors. GFP expression is homogenous between cells and titrates with lentivirus concentration. (TIF) [file pcbi.1006905.s003.tif]

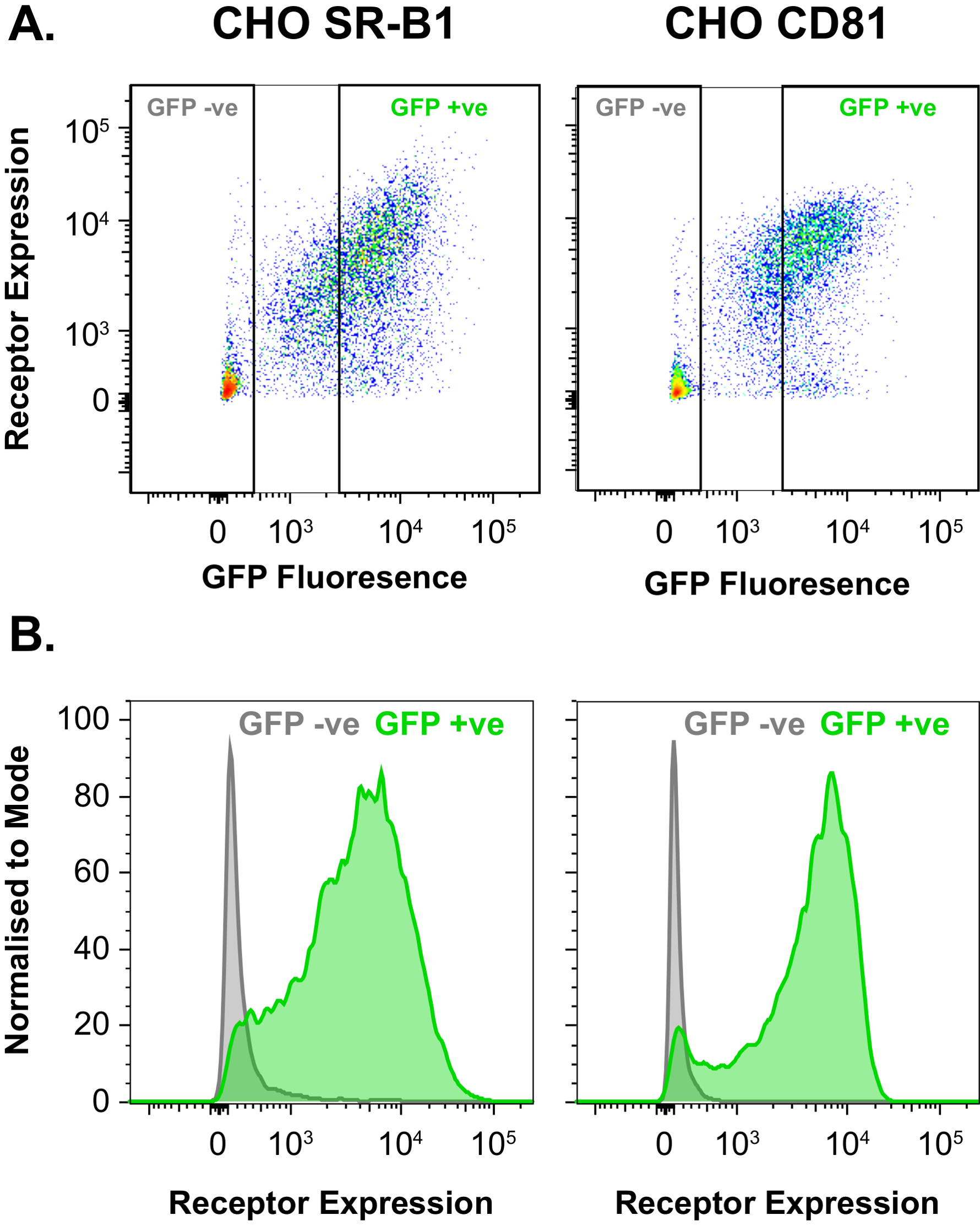

Supplement: S4 Fig — CHO cells were transduced with lentivirus encoding either SR-B1 or CD81 and GFP (as described in S3 Fig), receptor expression was assessed by flow cytometry. A. Representative dot plots of receptor and GFP expression in CHO cells, unlike Huh-7.5 cells, a minority of cells remained GFP/receptor negative. B. Representative histograms of receptor expression in GFP negative and positive CHO cells, as expected, receptor expression is only apparent in GFP positive cells. (TIF) [file pcbi.1006905.s004.tif]

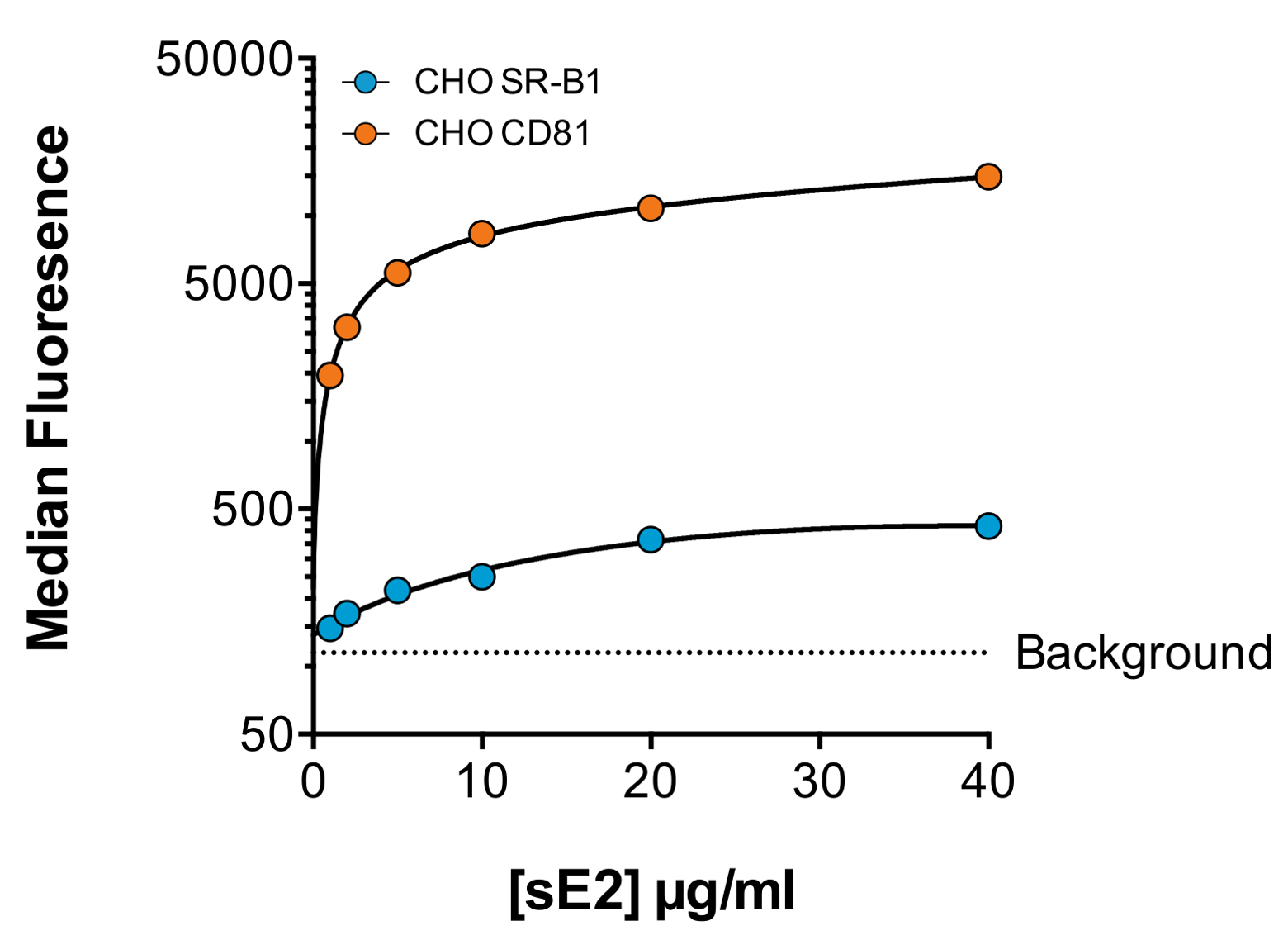

Supplement: S5 Fig — Representative median fluorescence intensity values for sE2 binding to CHO SR-B1/CD81 cells, as assessed by flow cytometry. Background is determined by sE2 binding to untransduced CHO cells. Data points represent the mean of n = 2 technical repeats. Error bars indicate standard error of the mean. Data was fitted using a one-site binding curve in GraphPad Prism. (TIF) [file pcbi.1006905.s005.tif]

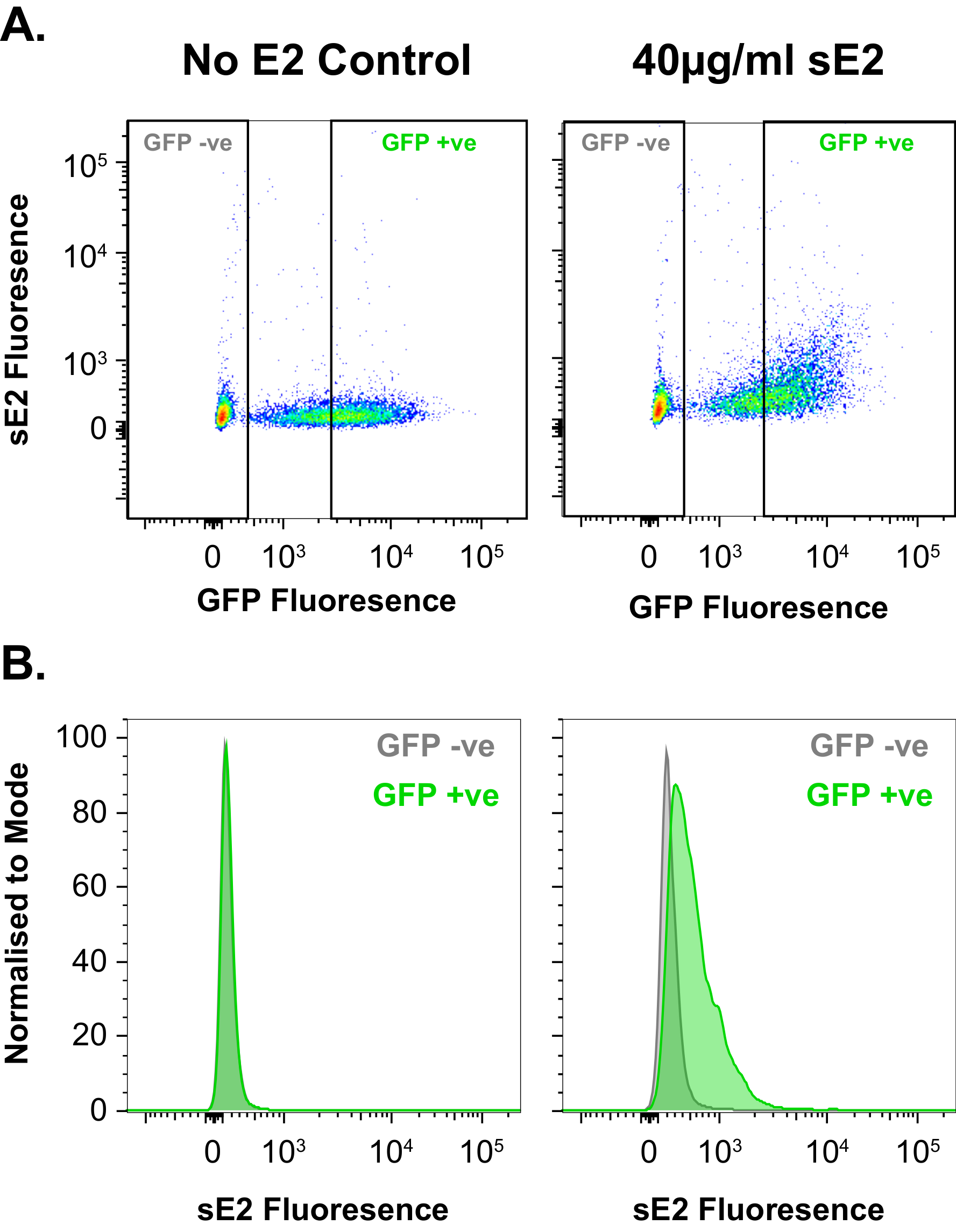

Supplement: S6 Fig — Representative raw data showing sE2 binding to CHO cells transduced with lentiviral vectors encoding CD81 + GFP. A. Dot plots displaying sE2 binding and GFP expression in untreated CHO-CD81 cells and those incubated with 40μg/ml sE2. B. sE2 binding to GFP negative and positive cells within the same sample, as expected, sE2 binding is only detectable in GFP positive cells, i.e those that have been successfully transduced with receptor encoding lentivirus. (TIF) [file pcbi.1006905.s006.tif]

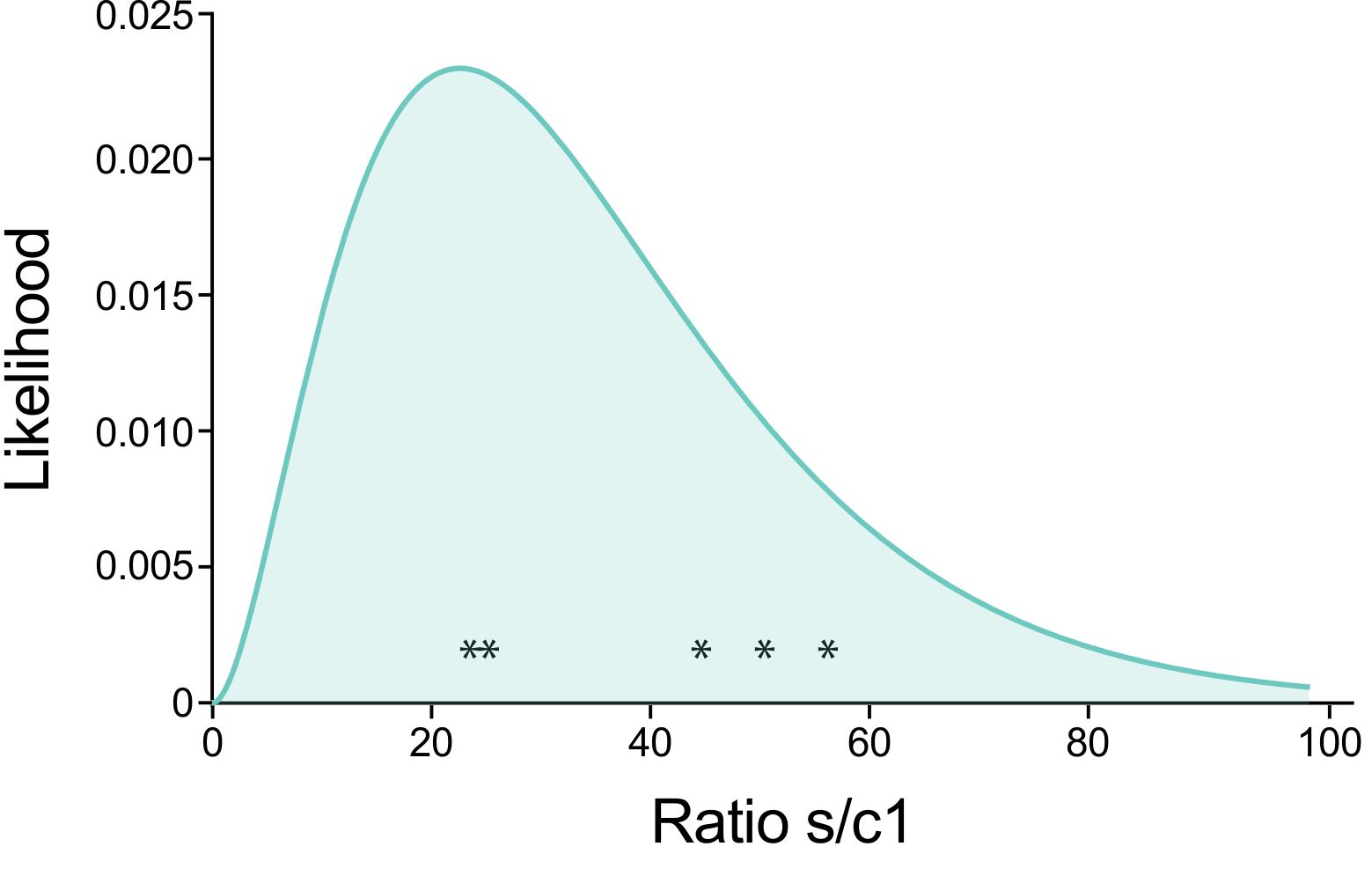

Supplement: S7 Fig — Data from the sE2 binding experiments (Fig 4) were used to characterise the ratio between the intrinsic binding of the virus to CD81 and SR-B1 receptors. A gamma distribution with parameters α and β, was fitted to data describing the ratio between the extent of viruses bound at saturation. Peak likelihood was achieved at a ratio of ~20. Asterisks indicate the experimentally measured ratio from 5 independent experiments. (TIF) [file pcbi.1006905.s007.tif]

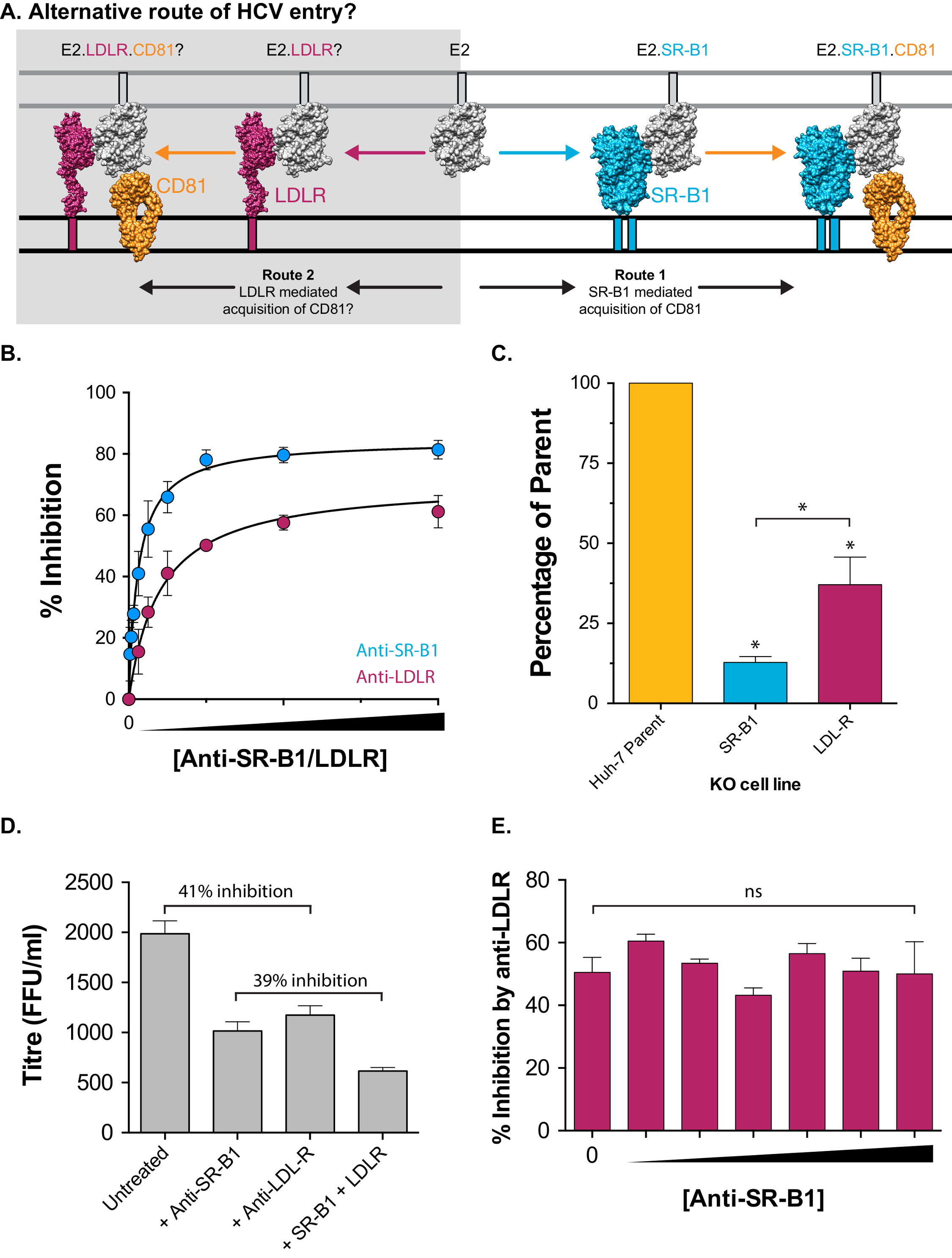

Supplement: S8 Fig — A. A report by Yamamoto et. al. suggests redundancy between SR-B1 and LDLR. Therefore we investigated the possibility that LDLR functions in an analogous manner to SR-B1, as illustrated. Note the molecular model of LDLR is based on a partial crystal structure (PDB: 3P5C [65]); full length LDLR is likely to be significantly longer than represented here. B. Antibody mediated receptor blockade of SR-B1 and LDLR inhibit HCVcc infection. C. Titre of HCVcc in Huh-7 cells gene-edited to remove SR-B1 and LDLR, data is expressed as a percentage of parental cells. Both gene edited cell lines display a statistically significant reduction in titre when compared to parental cells, and to each other, as denoted by asterisks. D. Example raw infection data upon combination of anti-SR-B1 and anti-LDLR. SR-B1 blockade does not increase the inhibitory activity of anti-LDLR, as annotated on the plot. E. Inhibition of HCV infection by anti-LDLR upon increasing concentration of anti-SR-B1, we measured no significant difference between treatments. This lack of synergy suggests that the model presented in A is incorrect; LDLR does not mediate SR-B1-independent entry. All data points are mean values of n = 3 independent repeats, apart from D., which displays raw data. Error bars indicate standard error of the mean. Significance testing performed using an unpaired t-test (GraphPad Prism). (TIF) [file pcbi.1006905.s008.tif]

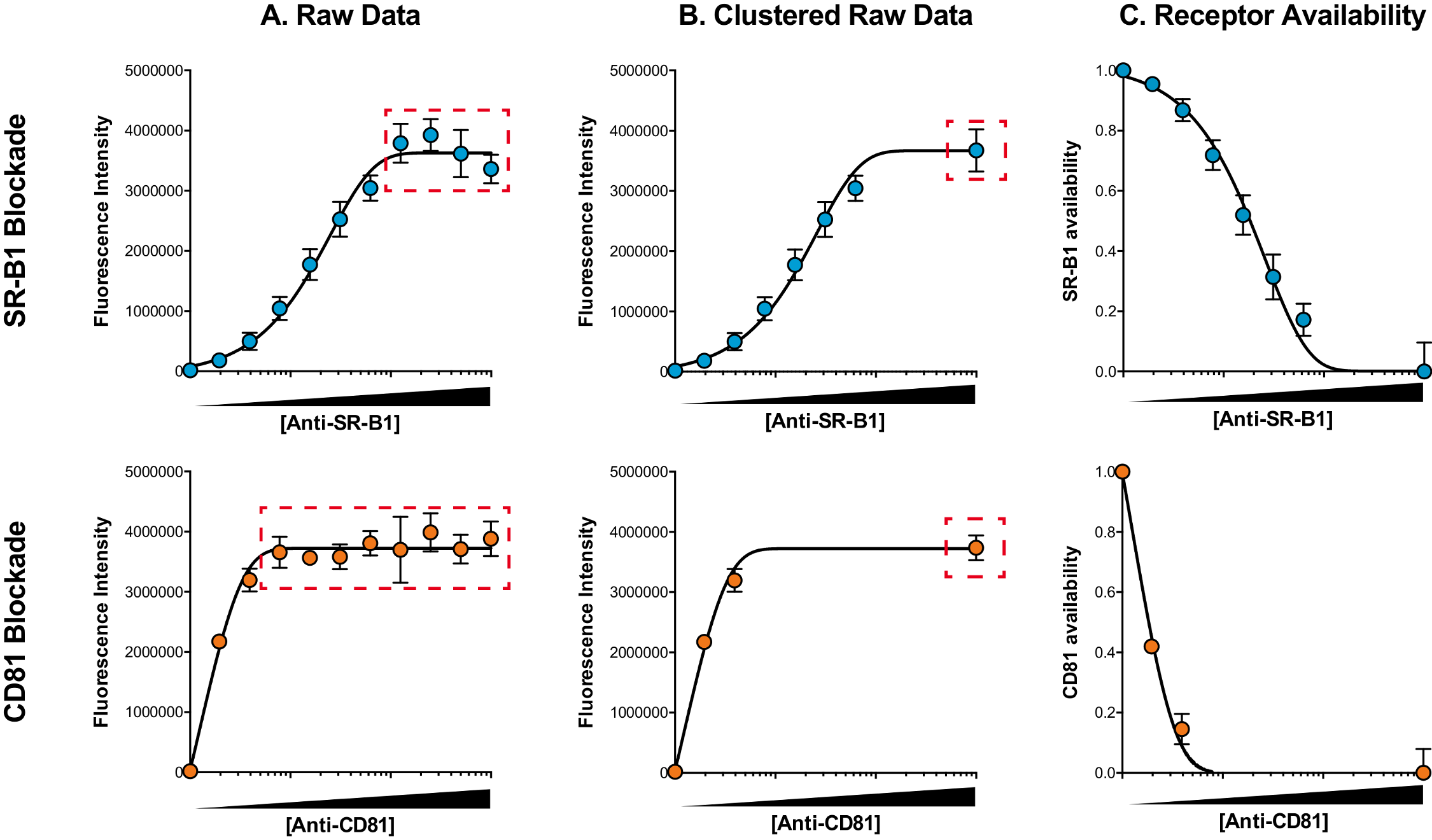

Supplement: S9 Fig — We estimated receptor availability from fluorescence microscopy data. A. Representative raw fluorescence measurements of anti-SR-B1/CD81 binding to Huh-7.5 cells (similar to Fig 2A), data points represent the mean of n>4 technical repeats, error bars indicate standard deviation of the mean. B. Statistically indistinguishable data points were clustered together and averaged; the clustered measurements and their resulting combined data point are annotated. C. Scaled estimates of receptor availability derived from clustered data. Maximum antibody binding indicates saturation of receptor and, therefore, availability = 0; whereas in untreated cells receptor availability is set to 1. These receptor availability values can then be compared to matched infection data to explore HCV receptor availability. In each plot data was fitted using a sigmoidal curve in GraphPad Prism. (TIF) [file pcbi.1006905.s009.tif]
