# Supplementary material for: Building a mechanistic mathematical model of hepatitis C virus entry
Source: PLoS Comput Biol. 2019 Mar 18;15(3):e1006905. doi: 10.1371/journal.pcbi.1006905 (PMC6445459; doi:10.1371/journal.pcbi.1006905)
Supplement: S1 Table — While the sE2 data constrain the ratio between the parameters s and c1, this had very little impact on the final optimised values. (DOCX) [file pcbi.1006905.s010.docx]

|  | **Primary Model : Complete likelihood** | **Primary Model : Without sE2 data** |
| --- | --- | --- |
| **Optimised parameters** |  |  |
| Intrinsic ability to acquire SR-B1 (s) | **0.58** | 0.58 |
| Intrinsic ability to acquire CD81 (c_1_) | **0.24** | 0.24 |
| Primed acquisition of CD81 (c_2_) | **1.36** | 1.36 |
| Rate of downstream viral entry (e) | **3.98 x 10^-4^** | 3.98 x 10^-4^ |
| **Predicted entry efficiency in unmodified cells** |  |  |
| Proportion of particles that acquire sufficient CD81 | **36.8%** | 36.7% |
| Proportion of particles that complete downstream events | **3.8%** | 3.8% |
| Proportion of particles that achieve infection | **1.4%** | 1.4% |
