# Supplementary material for: Building a mechanistic mathematical model of hepatitis C virus entry
Source: PLoS Comput Biol. 2019 Mar 18;15(3):e1006905. doi: 10.1371/journal.pcbi.1006905 (PMC6445459; doi:10.1371/journal.pcbi.1006905)
Supplement: S1 Text — We describe a null model, in which SR-B1 has no effect on viral entry, and models with additional parameters describing the loss of CD81 and SR-B1 receptors. (DOCX) [file pcbi.1006905.s011.docx]

## S1Text: Alternative models for evaluating viral entry

A range of alternative models to that described in the main text were evaluated, exploring different aspects of the data.

**No CHO model**

The ‘No CHO model’ was implemented to test the effect of the CHO data upon the optimisation and omitted the influence of the CHO data in constraining the ratio between the parameters *c_1_* and *s*. Whereas the model likelihood in the model presented in the main text was calculated as

$$\log L=\log L_{1}+\log L_{2}$$

Where *L_1_* is the likelihood arising from the fit to the viral entry data and *L_2_* is the likelihood arising from the fit to the CHO data, in this model the likelihood was simply calculated as

$$\log L=\log L_{1}$$

Results from this model showed that the CHO data had little impact upon the parameters inferred by our model. The requirement to fit the viral entry data dominated the requirement to fit the CHO data.

**Null model**

A null model was produced, describing a minimal model in which SR-B1 had no effect on binding to CD81. In this model, the state of a virus is characterised purely by the number of CD81 receptors that it has bound to, with the proportion of viruses to have bound *i* CD81 receptors being given by *M_i_*.

The model retains the basic properties of the original, albeit without the binding of SR-B1 receptors or the distinction between primed and un-primed E2 proteins.

Mathematically the model is given by

$$\frac{dD}{dt}=d\sum_{i} M_{i}$$

$$\frac{dE}{dt}=eM_{r}$$

$$\frac{dM_{i}}{dt}=\frac{1}{N_{e}}\left[ p_{c}c_{1}{\left( N_{e}-i+1 \right)M}_{i-1}-p_{c}c_{1}\left( N_{e}-i \right)M_{i} \right]-dM_{i} \mathrm{if} i<r$$

$$\frac{dM_{r}}{dt}=\frac{1}{N_{e}}\left[ p_{c}c_{1}{\left( N_{e}-r+1 \right)M}_{r-1}-p_{c}c_{1}\left( N_{e}-r \right)M_{r} \right]-dM_{r}-eM_{r}$$

where *M_i,j_* is defined as being zero for all *i* outside of the range 0 ≤ *i* ≤ *r.* Optimisation of the null model gave a substantially worse fit to the data than the model described in the main text, largely due to its failure to account for the effect of SR-B1 on viral entry.

**Unbinding of CD81**

A further model was tested, elaborating upon the original model by allowing unbinding of interactions between E2 and CD81 receptors. This was incorporated into the model by the addition of the parameter *c_3_*. The rate of unbinding was here assumed to be proportional to the number of E2-CD81 interactions that exist; that is, a single E2 protein unbinds a single CD81 receptor at a rate independent of anything else. As such, the full model was described by the equations:

$$\frac{dD}{dt}=d\sum_{ij} M_{i,j}$$

$$\frac{dE}{dt}=e\sum_{j=r} M_{i,j}$$

$$\frac{dM_{i,j}}{dt}=\frac{1}{N_{e}}\left[ p_{c}\left( c_{1}{\left( N_{e}-i-j+1 \right)M}_{i-1,j}+c_{2}{jM}_{i-1,j-1} \right)+p_{s}\left( s\left( N_{e}-i-j+1 \right)M_{i,j-1} \right) \right]-\frac{1}{N_{e}}\left[ \left( p_{c}\left( c_{1}\left( N_{e}-i-j \right)+c_{2}j \right)+p_{s}\left( N_{e}-i-j \right) \right)M_{i,j} \right]\boldsymbol{+}\boldsymbol{c}_{\boldsymbol{3}}\left[ \left( \boldsymbol{i+1} \right)\boldsymbol{M}_{\boldsymbol{i+1,j}}\boldsymbol{-i}\boldsymbol{M}_{\boldsymbol{i,j}} \right]-dM_{i,j} \mathrm{if} i<r$$

$$\frac{dM_{i,j}}{dt}=\frac{1}{N_{e}}\left[ p_{c}\left( c_{1}{\left( N_{e}-i-j+1 \right)M}_{i-1,j}+c_{2}{jM}_{i-1,j-1} \right)+p_{s}\left( s\left( N_{e}-i-j+1 \right)M_{i,j-1} \right) \right]-\frac{1}{N_{e}}\left[ \left( p_{c}\left( c_{1}\left( N_{e}-i-j \right)+c_{2}j \right)+p_{s}\left( N_{e}-i-j \right) \right)M_{i,j} \right]\boldsymbol{+}\boldsymbol{c}_{\boldsymbol{3}}\left[ \left( \boldsymbol{i+1} \right)\boldsymbol{M}_{\boldsymbol{i+1,j}}\boldsymbol{-i}\boldsymbol{M}_{\boldsymbol{i,j}} \right]-dM_{i,j}-eM_{i,j} \mathrm{if} i=r$$

where the additional component of the model is highlighted in bold text.

Optimisation of this model against the data gave values of *c_3_* very close to zero, indicating that the data did not support an unbinding term of this nature. Outputs from this model were therefore very close to those identified by the original model. We note that other models of the unbinding of CD81 receptors could potentially be considered.

**Unbinding of SR-B1**

A final model allowed the unbinding of interactions between E2 and SR-B1 receptors. This was incorporated into the model by splitting the parameter *s*, which described the rate of gain of SR-B1 receptors into *s_1_* and *s_2_*, indicating the rates of gain and loss of SR-B1 respectively. Similarly to the model of CD81 unbinding it was assumed that a single E2 protein unbinds a single SR-B1 receptor at a rate unaffected by anything else in the system. As such, the full model was described by the equations:

$$\frac{dD}{dt}=d\sum_{ij} M_{i,j}$$

$$\frac{dE}{dt}=e\sum_{j=r} M_{i,j}$$

$$\frac{dM_{i,j}}{dt}=\frac{1}{N_{e}}\left[ p_{c}\left( c_{1}{\left( N_{e}-i-j+1 \right)M}_{i-1,j}+c_{2}{jM}_{i-1,j-1} \right)+p_{s}\left( \boldsymbol{s}_{\boldsymbol{1}}\left( N_{e}-i-j+1 \right)M_{i,j-1} \right) \right]-\frac{1}{N_{e}}\left[ \left( p_{c}\left( c_{1}\left( N_{e}-i-j \right)+c_{2}j \right)+p_{s}\left( N_{e}-i-j \right) \right)M_{i,j} \right]+\boldsymbol{s}_{\boldsymbol{2}}\left[ \left( \boldsymbol{j+1} \right)\boldsymbol{M}_{\boldsymbol{i,j+1}}\boldsymbol{-j}\boldsymbol{M}_{\boldsymbol{i,j}} \right]-dM_{i,j} \mathrm{if} i<r$$

$$\frac{dM_{i,j}}{dt}=\frac{1}{N_{e}}\left[ p_{c}\left( c_{1}{\left( N_{e}-i-j+1 \right)M}_{i-1,j}+c_{2}{jM}_{i-1,j-1} \right)+p_{s}\left( \boldsymbol{s}_{\boldsymbol{1}}\left( N_{e}-i-j+1 \right)M_{i,j-1} \right) \right]-\frac{1}{N_{e}}\left[ \left( p_{c}\left( c_{1}\left( N_{e}-i-j \right)+c_{2}j \right)+p_{s}\left( N_{e}-i-j \right) \right)M_{i,j} \right]$$

$$+\boldsymbol{s}_{\boldsymbol{2}}\left[ \left( \boldsymbol{j+1} \right)\boldsymbol{M}_{\boldsymbol{i,j+1}}\boldsymbol{-j}\boldsymbol{M}_{\boldsymbol{i,j}} \right]-dM_{i,j}-eM_{i,j} \mathrm{if} i=r$$

where again the additional components of the model are highlighted in bold text. Optimisation of this model gave a slightly better fit to the data than the original model, albeit the additional parameter made a full optimisation computationally challenging. The biological picture created by this model is strongly different from that of the original model; the optimised rate of viral acquisition of SR-B1 was generally lower, and in some scenarios much lower, than the rate of unbinding of SR-B1. Further, the difference between the parameters *c_1_* and *c_2_* was much greater than that of the original model, creating a picture in which viruses fleetingly bind SR-B1, but while bound, gain CD81 receptors at a very rapid rate. While in itself, this could be a plausible mechanism for entry, the CHO data, which was here used only to constrain the ratio between *s_1_* and *c_1_*, suggests that SR-B1 is easily acquired (and consequently retained) by the virus, contradicting this inference. While this model provides a better likelihood than that obtained in the main text, we are therefore cautious about accepting its biological implications; more data are likely required to draw firm conclusions about the precise nature of virus-receptor interactions.
